# A Variant of TNFR2-Fc Fusion Protein Exhibits Improved Efficacy in Treating Experimental Rheumatoid Arthritis

**DOI:** 10.1371/journal.pcbi.1000669

**Published:** 2010-02-05

**Authors:** Tong Yang, Zheng Wang, Fang Wu, Jingwei Tan, Yijun Shen, Erguang Li, Jingzhi Dai, Ronghai Shen, Gang Li, Jinsong Wu, Luochun Wang, Haibo Wang, Yanjun Liu

**Affiliations:** 1 Department of Genetic Engineering Development, Shanghai Fudan-Zhangjiang Bio-pharmaceutical Co., Ltd., Shanghai, China; 2 School of Medicine, Nanjing University, Nanjing, China; 3 Shanghai Institute of Pharmaceutical Industry, Shanghai, China; 4 Taizhou Fudan-Zhangjiang Bio-pharmaceutical Co., Ltd., Taizhou City, Jiangsu Province, China; University of Houston, United States of America

## Abstract

Etanercept, a TNF receptor 2-Fc fusion protein, is currently being used for the treatment of rheumatoid arthritis (RA). However, 25% to 38% of patients show no response which is suspected to be partially due to insufficient affinity of this protein to TNFα. By using computational protein design, we found that residue W89 and E92 of TNFR2 were critical for ligand binding. Among several mutants tested, W89Y/E92N displayed 1.49-fold higher neutralizing activity to TNFα, as compared to that of Etanercept. Surface plasmon resonance (SPR) based binding assay revealed that the equilibrium dissociation constant of W89Y/E92N to TNFα was 3.65-fold higher than that of Etanercept. In a rat model of collagen-induced arthritis (CIA), W89Y/E92N showed a significantly better ability than Etanercept in reducing paw swelling and improvement of arthritic joint histopathologically. These data demonstrate that W89Y/E92N is potentially a better candidate with improved efficacy in treating RA and other autoimmune diseases.

## Author Summary

Rheumatoid arthritis (RA) is a frequently occurring, chronic, debilitating disease. TNFα plays a pivotal role in regulating its inflammatory response. TNFα inhibition with TNF receptor 2-Fc fusion protein (TNFR2-Fc) was effective in the treatment of RA. However, quite a few patients may not achieve good clinical outcomes after TNFR2-Fc therapy, and the high dosage of TNFR2-Fc in clinical treatment usually causes some side effects, such as injection site reactions. Enhancing the affinity of TNFR2-Fc to TNFα would be of benefit to its therapeutic effect on RA, and may reduce the clinical dosage. We modeled the interactions of TNFα and TNFR2, and figured out the sites that may be critical for ligand-receptor binding. A high affinity TNFR2-Fc variant (E92N/W89Y) was obtained by rational mutagenesis at residue 89 and 92. It shows significant improvements compared with wild type TNFR2-Fc in suppressing rat arthritis induced by collagen. This variant is more potent in neutralizing TNFα, and thereafter may offer a higher degree of RA symptom relief, and be in a much lower dosage.

## Introduction

Overproduction of TNFα is an underlying mechanism of autoimmune diseases, including RA, ankylosing spondylitis and psoriatic arthritis [1]–[2]. Blocking excess TNFa by its antagonists including TNF receptor 2-IgG1 fusion protein (Etanercept) and anti-TNFα monoclonal antibodies (Infliximab and Adalimumab) has been validated as an effective treatment for RA [1], [3]–[4], although not all patients respond well to the treatment (25% to 38% of Etanercept patients; 21% to 42% of Infliximab patients). Etanercept has been shown to be efficacious in a proportion of patients who did not respond to Infliximab, and vice versa [5]. The failure of Etanercept in some RA patients and in some autoimmune diseases, such as Crohn's disease, was likely due to its low affinity to TNFα [6]–[8]. A higher affinity TNFR2-Fc variant is believed to possess better efficacy than Etanercept.

We developed some higher affinity TNFR2-Fc variants by computational protein design method. Since the structures of TNFR2-TNFα complex and TNFR2 were absent, we chose 1a8m and 1tnr, the crystal structure of a TNFα variant and a complex of TNFR1 and TNFβ in protein data bank, as templates to model the interactions of TNFR2-TNFα. TNFα and TNFβ share high sequence identity and similar binding characters to two common receptors, TNFR1 and TNFR2, containing 4 highly conserved cysteine rich domains within the extracellular region [9]–[10]. We therefore constructed the TNFR2-TNFβ model by molecular modeling software package InsightII (Accelrys Inc.). According to the model, we found that the amino acid residues 89 and 92 of TNFR2 are critical for binding with TNFα and the corresponding mutants were expressed in a mammalian cell system (CHO-K1 cells). A mutant with combined mutation at these two sites, W89Y/E92N, displayed improved activity against TNFα in both *in vitro* and *in vivo* assays. Here we report the design and characterization of this high affinity TNFR2-Fc variant, and focus on evaluating its therapeutic effect of neutralizing activity of TNFα on RA.

## Results

### 

#### Residues W89 and E92 of TNFR2 are critical for ligand binding

**Figure 1:**
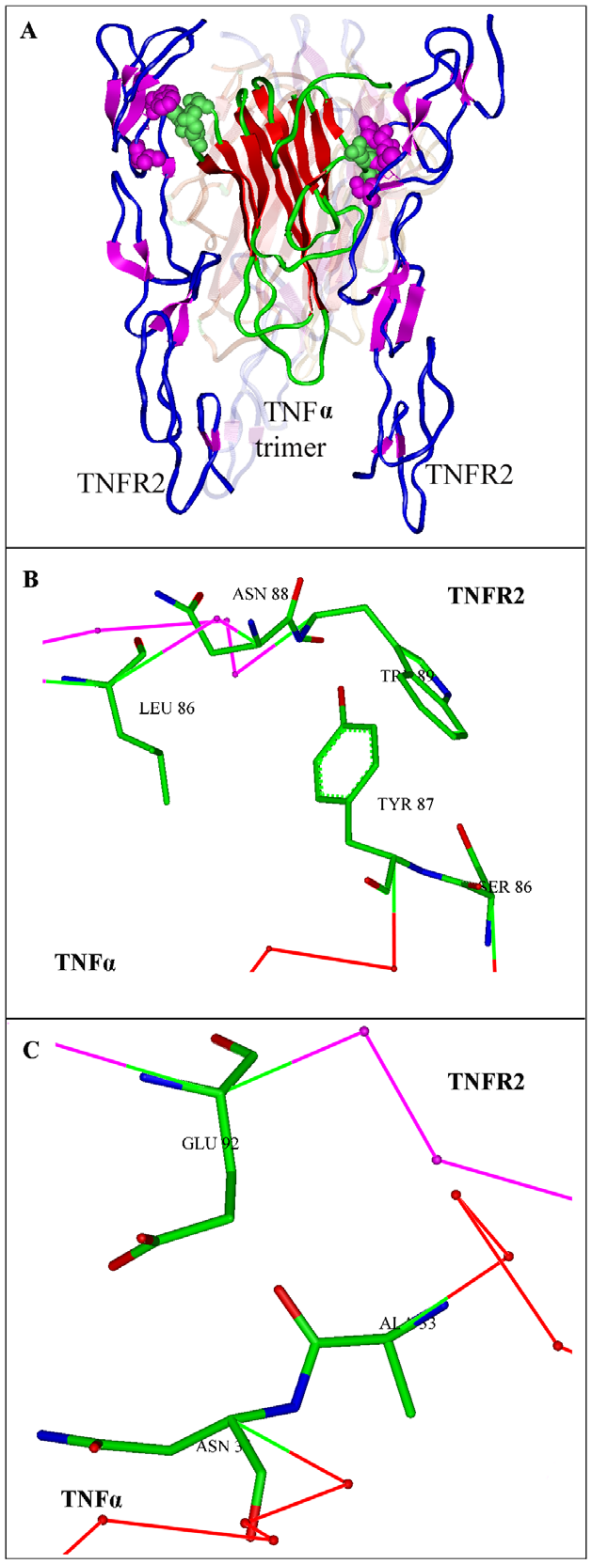
The overall structure of TNFR2-TNFα. The model was homologically modeled with the templates 1tnr and 1a8m, the TNFβ-TNFR1 complex and a TNFα variant crystal structure. (A) The model structure of TNFR2-TNFα complex. TNFR2 are shown using purple and blue cartoons, while TNFα is in red. (B) The specific interactions of residue W89 (TRP) of TNFR2 with TNFα. (C) The specific interactions of residue E92 (Glu) of TNFR2 with TNFα.

We modeled the TNFR2-TNFα complex with the template 1a8m and 1tnr by the computer program Homology, a module within InsightII, the backbone of the TNFR1 in 1tnr was substituted by the corresponding residues of TNFR2, the construction of disulfide bonds was referenced to Banner's models [10]–[11], and the overall structure of TNFβ in 1tnr was replaced by TNFα of 1a8m. The TNFR2-TNFα interactions were optimized by Amber force-field in the Discover module of InsightII. The TNFR2-TNFα model reveals that TNFR2 binds to TNFα mainly through the second cysteine rich domain, and residue W89 and E92 of TNFR2 is crucial for binding with loop 29–34 and 85–89 of TNFα. The sketch map of TNFR2-TNFα interactions is shown in Fig. 1 A, and the specific interactions of residue W89 and E92 of TNFR2 with TNFα are depicted in Fig. 1 B and C. Residue W89 and E92 appear to interact with Y87, and N34 of TNFα, respectively. The binding energy of W89 and 92 to the counterparts of TNFα also prompted their importance in ligand binding (data not shown). We therefore chose residues 89 and 92 for further mutagenesis studies.

#### TNFR2-Fc variants with residue 89 and 92 mutation displayed improved activity in neutralizing the cytotoxic effect of TNFα

Site-directed mutagenesis at amino acids 89 and 92 of TNFR2 was performed to determine the contribution of these residues to the ligand binding properties, and the effect of these variants on TNFα mediated cytotoxicity was characterized by the neutralizing activity assay. As shown in Table 1, mutants E92A, E92H and E92N, exhibited about 30% elevation of neutralizing activity on TNFα; while the neutralizing activity of mutant E92S decreased to only about 3% that of Etanercept; residue 89 substituted by H, F, I and M also impaired the neutralizing activity on TNFα greatly. Theses results confirmed the importance of residue 89 and 92 to binding with TNFα. By studying mutagenesis at 92 and 89 simultaneously, we generated W89Y/E92N, E92S/W89Y and E92N/W89F mutants and found that the combined mutations had enhanced neutralizing activity against TNFα. W89Y/E92N, the most potent variant in the neutralizing assay, was selected for further evaluation.

**Table 1:** The neutralizing activity of Etanercept and its variants on TNFα-induced cytotoxicity in mouse L929 cells.

TNFR2-Fc proteins	Neutralizing activity against TNFα (Unit/mg)^a^
Etanercept	1.62×10^6^
W89H	4.14×10^5^
W89F	1.23×10^6^
W89I	<2.00×10^4^
W89Y	1.32×10^6^
W89M	4.00×10^5^
E92A	2.10×10^6^
E92S	4.86×10^4^
E92H	2.09×10^6^
E92N	2.12×10^6^
W89Y/E92N	2.42×10^6^
W89Y/E92S	2.16×10^6^
W89F/E92N	2.07×10^6^

a 1 unit is the dose of Etanercept that neutralised the cytotoxic activity of 1 IU TNFα on mouse L929 cells. Values are means of a triplicate experiment.

#### W89Y/E92N mutant exhibits higher binding affinity for TNFα

The binding kinetics data of the W89Y/E92N and TNFR2 was obtained using the SPR technique (Table 2). Compared with Etanercept, the equilibrium dissociation constant (***K_D_***) of W89Y/E92N for TNFα showed a 3.65-fold decrease, which meant the affinity of W89Y/E92N to TNFα was enhanced. The binding kinetics indicates that the ligand binding mode of W89Y/E92N might differ from that of Etanercept. Specifically, the dissociation kinetic constant (***k_d_***) of W89Y/E92N for TNFα (1.29×10^−6^ s^−1^) was markedly lower than that of Etanercept (4.1×10^−6^ s^−1^); while the association kinetic constant (***k_a_***) of W89Y/E92N (9.72×10^3^ M^−1^s^−1^) for TNFα was only a little higher than that of Etanercept (8.45×10^3^ M^−1^s^−1^). Therefore, we identify W89Y/E92N a higher affinity TNFR2-Fc variant, and take notice of its lowered dissociation rate to TNFα compared to Etanercept, which may improve its pathologic effect *in vivo*.

**Table 2:** The binding kinetics profiles of the Etanercept and W89Y/E92N to TNFα obtained by SPR.

Interaction	k_a_ a (M^−1^s^−1^)	k_d_ b (s^−1^)	K_D_ c (nM)
TNF-Etanercept	8.45×10^3^	4.10×10^−6^	0.485
TNF-W89Y/E92N	9.72×10^3^	1.29×10^−6^	0.133

a Association kinetic constant.

b Dissociation kinetic constant.

c Equilibrium dissociation constant. The SPR data were analyzed by BIAevaluation software version 3.0.

#### W89Y/E92N possesses enhanced efficacy for the treatment of CIA in rats

**Figure 2:**
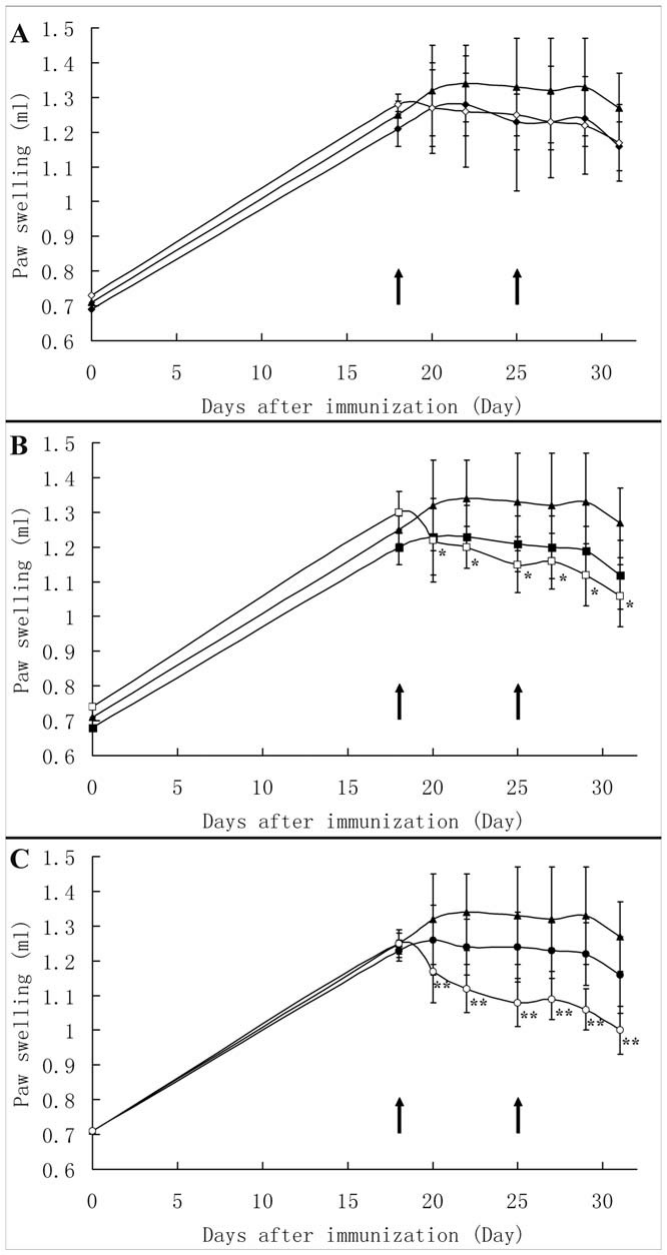
Paw-swelling. (A) Comparison of 1mg/kg Etanercept treated vs. the same dose of W89Y/E92N. (B) Comparison of 3mg/kg Etanercept treated vs. the same dose of W89Y/E92N. (C) Comparison of 9mg/kg Etanercept treated vs. the same dose of W89Y/E92N. Arrows indicate the time of treatment. *, **, a significant reduction of paw swelling of W89Y/E92N treatment compared to the groups given Etanercept (P<0.05; P<0.01 student t test). There were 10 rats per group. Black triangle, untreated group; Black diamond, 1mg/kg Etanercept treated group; White diamond, 1mg/kg W89Y/E92N treated group; Black square, 3 mg/kg Etanercept treated group; White square, 3mg/kg W89Y/E92N treated group; Black circle, 9 mg/kg Etanercept treated group; White circle, 9mg/kg W89Y/E92N treated group.

The *in vivo* effect of W89Y/E92N and Etanercept was evaluated by employing a rat model of CIA, and the paw swelling of rats was monitored throughout the period of arthritis by drainage method [12]. W89Y/E92N and Etanercept treatment reduced the paw swelling induced by collagen compared to the normal saline treatment (untreated group). Treatment with W89Y/E92N resulted in a dose-dependent reduction in paw swelling over the treatment course (Fig. 2), with doses of 3 and 9 mg/kg giving statistically significant reductions in paw swelling relative to rats given the same dose of Etanercept (P<0 05 and 0.01, respectively). After two week therapy, Etanercept treatment at 1, 3 and 9 mg/kg lowered paw swelling than the untreated group by 11.6, 15.2 and 16.2%, respectively, while the same dose of W89Y/E92N treatment reduced paw swelling by 20.9, 35.3 and 40.0% (Table 3). The reduction of paw swelling rate following 3mg/kg and 9mg/kg of W89Y/E92N treatment was 2.32 (35.3% vs 15.2%) and 2.47 (40.0% vs 16.2%) fold higher than that of the same dose of Etanercept treatment.

**Table 3:** The alleviation effect of W89Y/E92N and Etanercept treatment on paw swelling in the rat model of CIA.

Treatment	The paw swelling on day 0 post-immunization (ml)	The paw swelling on day 31 post-immunization (ml)	Increase (%)
Normal Saline	0.71±0.04	1.27±0.07	80.4±13.2
1 mg/kg Etanercept	0.69±0.05	1.16±0.08	68.8±13.1
3 mg/kg Etanercept	0.68±0.05	1.12±0.11	65.2±15.4
9 mg/kg Etanercept	0.71±0.03	1.16±0.09	64.2±16.3
1mg/kg W89Y/E92N	0.73±0.03	1.17±0.11	59.5±14.5
3 mg/kg W89Y/E92N	0.74±0.06	1.06±0.07	45.1±13.2^*^
9 mg/kg W89Y/E92N	0.71±0.03	1.00±0.10	40.4±14.9^**^

Rats were immunized with CII, and at day 18 after the first immunization, rats that showed RA symptom were subcutaneously treated with normal saline (untreated control group), 1, 3 and 9 mg/kg of Etanercept and W89Y/E92N, respectively, once per week for two consecutive weeks. Values are means±SD of ten rats for each group. Differences between groups were examined for statistical significance by using student t test. *, P<0.05, **, P<0.01 compared with the same dosage of Etanercept treatment.

**Figure 3:**
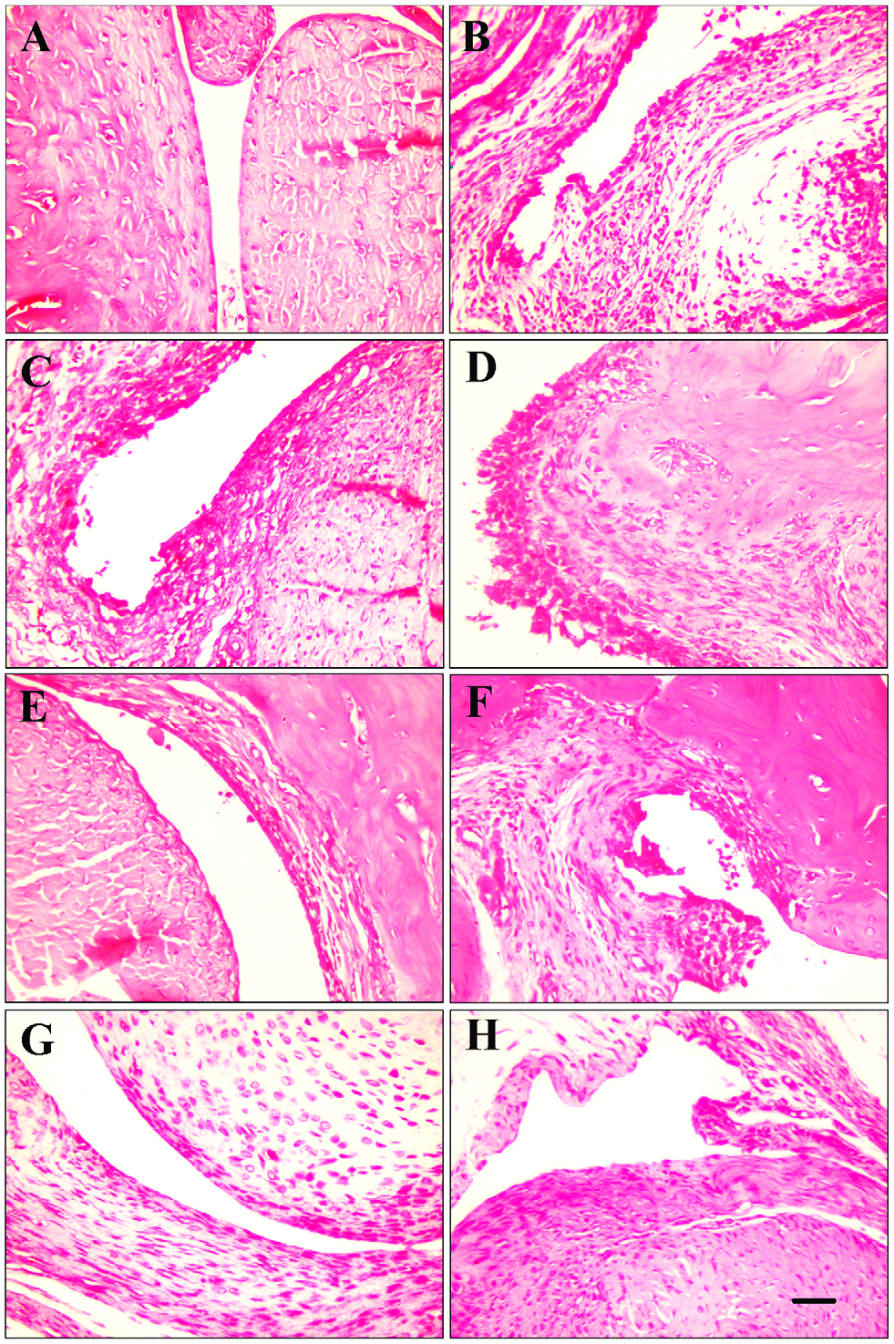
Representative joint histopathology of the groups with CIA administered with W89Y/E92N and Etanercept. Tissue sections from each group were stained with hematoxylin and eosin (HE). (A) None-immune group. (B) Untreated group treated with normal saline. (C) W89Y/E92N 1mg/kg treated group. (D) Etanercept 1mg/kg treated group. (E) W89Y/E92N 3mg/kg treated group. (F) Etanercept 3mg/kg treated group. (G) W89Y/E92N 9mg/kg treated group. (H) Etanercept 9mg/kg treated group. Scale shown on the right-hand side of (H) is equivalent to 0.1 mm.

The histopathologic changes of paw joints following W89Y/E92N and Etanercept treatment in rats suffering from CIA was shown in Fig 3. Characteristic of arthritic joints in rat CIA is synovial hyperplasia, pannus formation, exudation of cells into the joint space, and erosion of bone and cartilage [13]. A massive influx of inflammatory cells, synovial hyperplasia, and accumulation of abundant monomorphonuclear and polymorphonuclear cells in the joint space were evident in the untreated control group (Fig. 3 B) compared with non-immune group (Fig. 3 A). A reduced degree of arthritis severity was observed in the rats that received W89Y/E92N and Etanercept. By comparison, W89Y/E92N treated group showed better joint histopathology amelioration than the group treated with the same dosage of Etanercept. Arthritic rats treated with 9mg/kg of W89Y/E92N showed the minimal level of inflammation and joint destruction; specifically, the synovial membrane in the joints looked like normal synovium, with few signs of synovial hyperplasia or other characteristics of inflammation (Fig 3 G). The pathologic improvement by W89Y/E92N treatment indicates that this high affinity variant is a potentially alternative of Etanercept in RA therapy.

## Discussion

Computational protein design method has been demonstrated to represent a valuable tool for the improvement and modification of protein-protein interactions [14]–[15]. Etanercept is a dimeric molecule that is 50- to 1000-fold more potent than a monomeric form on the inhibition of TNFα activity in vitro, and thereafter reaches the required bioactivity for proper therapeutic activity [16]–[18]. Etanercept and its variants need to be prepared by the eukaryocyte expression system and can not be screened by some regularly high throughput methods. Modeling-guided specific mutagenesis of interacting residues enables the protein evolution in a limited region, and thereby accelerates the development of candidates with potent activity.

The extracellular part of TNFR2 shared about 30% sequence identity with TNFR1 and the alignment contains a long insertion and some deletions between the CRD3 to CRD4 (data not shown), it was difficult to model the whole structure of TNFR2 with TNFR1 accurately. However, The CRD2 and CDR3 of TNFR2 showed a considerable degree of sequence identity with TNFR1 (∼50%), consequently it was decided to model TNFR2 with the backbone of TNFR1 in 1tnr, and focus on the specific interactions of CDR2 and CDR3 of TNFR2 with its ligands. The TNFR2-TNFα model showed that residue 89 and 92 of TNFR2 may affect its binding to TNFα. The following site-directed mutagenesis found that residue substitution at these points could obtain potential candidates. We selected the variant W89Y/E92N for further evaluation, which displayed improved anti-TNFα activity in vitro. Consistent with results from neutralizing activity assays, we found that W89Y/E92N variant had higher affinity to TNFα than Etanercept, and had better pathologic improvement on experimental RA *in vivo*.

Etanercept is known to have fast rates of association and dissociation with TNFα. It releases TNFα within approximately 10 minutes, and again binds TNFα immediately [19]. When receptor fusion concentration is relatively high, those released TNFα will quickly reassociate with the receptor fusion and leave rarely free in the system. However, under situations where TNFα is released from the receptor fusion molecule and there are high numbers of cell associated TNFα receptors present and a lower level of receptor fusion (due to poor penetration), TNFα might bind to the cell-associated TNFR1 or TNFR2 instead of back onto the receptor fusion [20]. Thus it may be expected that W89Y/E92N would have better neutralizing activity on TNFα *in vivo* for its lowered dissociation rate compared with Etanercept (1.29×10^−6^ s^−1^ versus 4.10×10^−6^ s^−1^).

W89Y/E92N exhibited significant improvement in suppressing rat arthritis induced by collagen, presented lower paw swelling after two weeks treatment than the same dosage of Etanercept and better joint histopathology amelioration. The *in vivo* improvement of W89Y/E92N may considerably ascribe to its high affinity, as the pharmacokinetics profile of W89Y/E92N was comparable to that of Etanercept (data not shown). The fact that only some RA patients (about 40% Etanercept patients receiving 25 mg Etanercept twice weekly for 24 weeks and 38.9% of patients receiving MTX-6 mg/kg-Infliximab for 54 weeks) [20]–[21] showed a 50% improvement in the American College of Rheumatology Index makes a continued search for better therapeutants essential. There was growing evidence that the efficacy of anti-TNFα therapies in Crohn's disease may critically depend on the affinity of TNFα antagonists to TNFα [6]–[8]. Etanercept bound to TNFα with a 4-fold lower affinity with respect to Infliximab, which was believed to lead to the outcome that Crohn's disease patients responded to Infliximab but Etanercept [22]. The affinity of W89Y/E92N to TNFα was close to that of Infliximab, and thereby could have improved efficacy.

## Materials and Methods

### 

#### Modeling of TNFR2-TNFα complex

The crystal structure of TNFβ in complex with the TNFR1 is known as 1tnr in protein data bank (PDB) [10], and the crystal structure of a TNFα variant trimer as 1a8m in PDB. Data for 1tnr and 1a8m were used as template to model the three-dimensional structure of complex of TNFR2-TNFα using the computer program Homology of InsightII (Accelrys software Inc.). The backbone of the TNFR1 in 1tnr was substituted by the corresponding residues of TNFR2, the construction of disulfide bonds was referenced to Banner's models [10]–[11], and the overall structure of TNFβ in 1tnr was replaced by TNFα of 1a8m. The TNFR2-TNFα was refined with Discover in Amber Force field. To that end, TNFR2-TNFα model was optimized by applying 500 cycles of the steepest descent method followed by 500 cycles of the conjugate gradient method, respectively.

#### Generation of TNFR2-Fc mutants by site-directed mutagenesis

Amino acid substitutions of TNFR2-Fc were generated by PCR-based site-directed mutagenesis [23]. Two pairs of primers were desired for overlapping PCR: the forward primer (5′- aagcttATGGCTCCCGTCGCCGTCTGGG) comprising Hind III restriction site prior to the ATG start codon and a 24- to 36-mer reverse primer within TNFR2-Fc coding region containing the desired mutation, another pair was the forward primer complementary to the above reverse primer and the reverse primer (5′-gaattcctatttacccggagacaggg) comprising EcoRI restriction site at the end of Fc coding sequence. The PCR products of TNFR2-Fc mutants were digested with the Hind III and EcoRI, and inserted into the pcDNA3 (Invitrogen).

#### Expression and purification of mutants

Plasmids were amplified in the *E. coli* strain Top10F' and extracted by a commercial purification kit (Qiagen) following the manufacturer's instructions. CHO-K1 cells were transfected with a linear vector encoding a TNFR2-Fc variant using lipofectamine 2000 based protocol (Invitrogen). Stable transfectants were selected by culturing in a medium containing Geneticin (G418, Sigma) and selected clones were used for TNFR2-Fc protein production. The proteins were purified by Protein A affinity chromatography. The purity of recombinant proteins was analyzed by SDS-PAGE, and the protein concentrations were determined by Bradford method [24].

#### The neutralizing assay on TNFα-induced cytotoxicity in mouse L929 cells

Mouse L929 cells were seeded into 96-well microtiter plates at a density of 1.5×10^4^ cells/well in 100 µL DMEM supplemented with 10% FBS. The cells were preserved at 37°C for 18h. 0.1ml 20units/ml TNFα (Sigma) in present of 2 µg/ml actinomycin D (Sigma) was added per well, and then the cells was incubated with gradient concentrations of Etanercept (Amgen), TNFR2-Fc mutants or medium control for 24h at 37°C. Cell viability was measured by using a crystal violet staining method [25]. All assays were performed in triplicate.

#### Surface plasmon resonance assay

The binding kinetics of the Etanercept and W89Y/E92N for TNFα were analyzed by the surface plasmon resonance technique (BIAcore® 3000, G.E. Inc.). Etanercept and W89Y/E92N were immobilized onto a CM5 sensor chip (G.E. Inc.), which resulted in an increase of 5000–5500 resonance units (RU). During the association phase, TNFα diluted in running buffer (HBS-EP, 10 mM HEPES, 150 mM NaCl, 3 mM EDTA, 0.005% (v/v) surfactant P20, pH 7.4, Sigma) at 20.0, 10.0, 5.0, 2.5, 1.25, 0.625, 0.3125 nM, was allowed to pass over the chips immobilized with Etanercept and W89Y/E92N, respectively, at a flow rate of 40 µl/min for 1 min. During the dissociation phase, HBS-EP buffer was applied to the sensor chip at a flow rate of 40 µl/min for 2 min. The data were analyzed globally with the BIAEVALUATION 3.0 software (BIAcore®) using a 1∶1 Langmuir binding model [26].

#### The rat model of CIA

To assay for anti-TNFα inflammation activity *in vivo*, a CIA assay was performed as described [27]–[28]. In this model, female Wistar rats of approximately 8 weeks old, weighing 150–180g, were obtained from Shanghai Laboratory Animal Commission. (Shanghai, China).The animals were acclimated to the holding room for at least 7 days under the standard condition with free access to food and water before initiation of the studies. For the induction of CIA, the type II collagen (CII) (Sigma) was dissolved in 0.1M acetic acid (Sigma) overnight, CII and incomplete Freund's adjuvant were homogenized at a 1∶1 ratio to a final concentration of 1 mg/ml. The mixture was stirred at 4°C for overnight until the CII was completely emulsified. Each rat was injected with 0.1ml emulsion per site intradermally at 4 sites on the back and one site on the tail tip with a total of 0.5 ml (day 0). This immunization protocol was repeated one more time seven days post the first immunization (day 7). Induction and severity of arthritis was determined by change in ankle weight, measured using calipers. At day 18 after the first CII immunization, 70 rats that showed RA symptom were chosen and randomly distributed to 7 groups, 10 rats per group. Treated the inflammatory comparison group (untreated control group) with normal saline, the other groups with 1, 3, 9 mg/kg W89Y/E92N and 1, 3, 9mg/kg Etanercept (Amgen), respectively. Besides randomly chose ten rats that had not been immunized by CII as the blank control group, and treated this group only with normal saline. The administration of Etanercept and W89Y/E92N is once subcutaneous per week for two consecutive weeks. The right hind paw swelling was monitored by drainage with YLS-7A calipers (Beijing Anjideer Inc., China) [12],[29]. At last, these rats were executed, and their right hind paw joints were prepared for pathology analysis.
